# Preparation and Characterization of ZnO Nanoparticles Supported on Amorphous SiO_2_

**DOI:** 10.3390/nano7080217

**Published:** 2017-08-10

**Authors:** Ying Chen, Hao Ding, Sijia Sun

**Affiliations:** Beijing Key Laboratory of Materials Utilization of Nonmetallic Minerals and Solid Wastes, National Laboratory of Mineral Materials, School of Materials Science and Technology, China University of Geosciences (Beijing), Beijing 100083, China; chenying@cugb.edu.cn (Y.C.); 1012122105@cugb.edu.cn (S.S.)

**Keywords:** amorphous SiO_2_, load, monodisperse, ZnO nanoparticle, antibacterial

## Abstract

In order to reduce the primary particle size of zinc oxide (ZnO) and eliminate the agglomeration phenomenon to form a monodisperse state, Zn^2+^ was loaded on the surface of amorphous silica (SiO_2_) by the hydrogen bond association between hydroxyl groups in the hydrothermal process. After calcining the precursors, dehydration condensation among hydroxyl groups occurred and ZnO nanoparticles supported on amorphous SiO_2_ (ZnO–SiO_2_) were prepared. Furthermore, the SEM and TEM observations showed that ZnO nanoparticles with a particle size of 3–8 nm were uniformly and dispersedly loaded on the surface of amorphous SiO_2_. Compared with pure ZnO, ZnO–SiO_2_ showed a much better antibacterial performance in the minimum inhibitory concentration (MIC) test and the antibacterial properties of the paint adding ZnO–SiO_2_ composite.

## 1. Introduction

Antimicrobial tests and environmental toxicity tests have been widely explored in order to improve health, safety, and the environment [1,2,3]. Zinc oxide (ZnO), as a semiconductor material with a band gap of 3.3 eV at room temperature [4], has high chemical stability, strong photosensitivity and non-toxicity property and is widely used in antibacterial materials [5]. Compared with ordinary ZnO powder, ZnO nanoparticles have a large specific surface area and small size effect, and show wide application potential in microbial inhibition and mildew removal [6,7].

However, like most of the nanoparticles, ZnO nanoparticles are prone to forming serious agglomeration, including hard agglomeration among the particles formed via the chemical reaction of the surface groups and soft agglomeration formed by other physical effects [8]. It is difficult to depolymerize the particles involved in hard agglomeration. Therefore, the apparent grain size of the primary ZnO particles tends to increase to the micron scale and the normal performance of ZnO nanoparticles is inhibited. In the preparation process of ZnO nanoparticles, in addition to the control of the ZnO morphology and primary particle size, the agglomeration phenomenon of ZnO particles should be suppressed to obtain dispersed nanoparticles. Wang et al. synthesized the doped ZnO nanoparticles with the mixture of alcohol and water as the solvent according to a precipitation method [9]. Chen et al. prepared ZnO nanocrystals via the reaction of zinc stearate with excessive alcohol in the hydrocarbon solvent [10]. Weller et al. used the low-temperature solvent thermal method to synthesize dispersible spherical ZnO nanoparticles and nano-rods with zinc acetate as precursors in methanol [11]. However, these methods have low synthesis performance and limited control ability. Especially, the solvent thermal process [12,13] is required to deal with organic solvents and it is difficult to realize industrial production. Therefore, some nanoparticles (such as titanium dioxide, TiO_2_) [14,15] are supported on the surface or pores of the inorganic carrier. In this way, the strong interaction between the carrier surface and nanoparticles, and the forced isolation among the carrier particles efficiently prevent the agglomeration among the nanoparticles and improve the dispersion effects and functions.

Amorphous SiO_2_, commercially known as white carbon black, is an aggregate of SiO_2_ particles (SiO_2_·nH_2_O) and commonly used as a rubber reinforcing additive [16,17]. The primary particle size of SiO_2_ particles is generally 10–100 nm. SiO_2_ particles containing rich Si–OH groups, which can form a strong interaction between the SiO_2_ carrier surface and Zn–OH (precursors of ZnO). This interaction reduces the combination between Zn–OH and prevents its aggregation, thus contributing to the formation of monodisperse ZnO nanoparticles. In addition, small amorphous SiO_2_ particles have high dispensability and can prevent further agglomeration of ZnO–SiO_2_ composite. Therefore, amorphous SiO_2_ was selected as the carrier of supported ZnO nanoparticles.

Based on the above results, in this paper, the environmentally friendly hydrothermal method was adopted to prepare ZnO–SiO_2_ by loading Zn^2+^ on the surface of amorphous SiO_2_ and calcining active products at high temperatures. Moreover, the structures and antibacterial properties of as-prepared ZnO–SiO_2_ were explored.

## 2. Experimental Procedure

### 2.1. Materials

**Figure 1:**
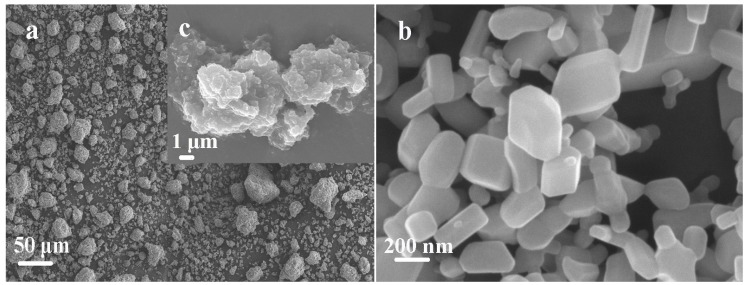
Micrographs of (**a**,**c**) amorphous SiO_2_ and (**b**) pure ZnO.

In this study, amorphous SiO_2_ was purchased from Henan Jiaozuo Fluoride New Energy Technology Co., Ltd (Jiaozuo, Henan, China). The properties of amorphous SiO_2_ are described as follows: SiO_2_ content of 96.63%, whiteness of 96.76%, average aggregate size of 20 μm, primary particle size of 20–30 nm, and specific surface area of 59.54 m^2^/g. Zinc nitrate (Zn(NO_3_)_2_·6H_2_O) as the source of Zn^2+^ was from Beijing Yili Fine Chemical Co., Ltd (Beijing, China). Sodium polyacrylate (PAAS) as a dispersant was supplied by Changzhou Run Yang Chemical Co., Ltd (Changzhou, Jiangsu, China). Pure ZnO, as an antibacterial agent, was compared with the ZnO–SiO_2_ composite in antibacterial performance. It was produced by the Xi Long Chemical Co., Ltd (Guangzhou, Guangdong, China) and the size of the particles was about 200 nm. Figure 1 shows SEM images of amorphous SiO_2_ and pure ZnO.

### 2.2. ZnO–SiO_2_ Precursor

**Figure 2:**
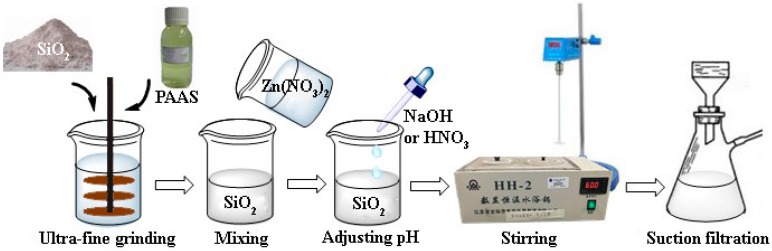
Preparation of composite particles of ZnO–SiO_2_ precursor.

Amorphous SiO_2_, sodium polyacrylate (1% of the weight of SiO_2_) and H_2_O were mixed and stirred to prepare the suspension with solid content of 18%. Ceramic polishing balls (diameter: 1–3 mm) were added into the suspension according to the proportion of 50% of the solid content and then stirred at a speed of 1000 r/min for 1 h to prepare the depolymerized amorphous SiO_2_ slurry. A zinc nitrate solution (0.09 wt %) was added into the slurry and the pH of the mixture was respectively adjusted to 5.0 and 7.0 by adding 6 mol/L NaOH and 6 mol/L HNO_3_. The mixture was stirred at 60 °C for 1 h. The precursors were obtained after suction filtration, washing, and drying and denoted as Zn–SiO_2_-pH5.0 (the precursor was prepared with pH value at 5.0) and Zn–SiO_2_-pH7.0 (the precursor was prepared with pH value at 7.0) respectively. The preparation process is shown in Figure 2.

### 2.3. Preparation of ZnO–SiO_2_

The precursors Zn–SiO_2_-pH5.0 and Zn-SiO_2_-pH7.0 were calcined at 400 °C for 1 h to obtain the composite particles of ZnO and SiO_2_ and denoted as ZnO–SiO_2_-pH5.0 (the composite obtained by calcining the precursor which was prepared with pH value at 5.0) and ZnO–SiO_2_-pH7.0 (the composite obtained by calcining the precursor which was prepared with pH value at 7.0). The loads of ZnO were 4.51 and 11.26%, respectively.

### 2.4. Characterization

The X-ray diffraction (XRD) was measured by using a D/max-Ra X-ray diffractometer (Ouyatu, Japan, Cu Kα radiation = 1.54 Å) in an angular range of 10–80° (2θ) with a step of 0.02° (2*θ*). Scherer Equation [18] is used to calculate the average grain size of ZnO nanoparticles:

(1) $$\beta =\frac{Κ\lambda }{Dcos\theta^{\prime \prime }}$$

where *K* is the shape factor constant (0.94); *λ* is X-ray wavelength; *D* is the grain size; *θ* is the diffraction angle; *β* is the diffraction peak half width.

An X-ray fluorescence spectrometer (XRF Shimadzu-1800, Kyoto, Japan) was used to analyze the oxide content of samples. The particle size and size distribution of the composite particles of ZnO and SiO_2_ were characterized by transmission electron microscopy (TEM FEI Tecnai G220, Portland, OR, USA). Scanning electron microscopy (SEM) was used to explore the morphology of ZnO–SiO_2_ by a Hitachi field emission scanning electron microscope (Hitachi S4800, Tokyo, Japan) under the voltage of 10 kV. The Fourier transform infrared spectroscopy (FTIR, Madison, WI, USA) measurement was carried out to explore the changes in functional groups of ZnO-SiO_2_ by Nicolet IS50. The samples were finely pulverized and then diluted in dried KBr to form a homogeneous mixture according to the sample-KBr ratio of 1/200. The X-ray photoelectron spectroscopy (XPS, Manchester, UK) measurement was conducted on an Axis Ultra spectrometer with monochromatic Mg Kα (1253.6 eV) radiation to investigate the valence state of Zn.

### 2.5. Antimicrobial Test

The antimicrobial ability of ZnO–SiO_2_ under dark conditions was investigated through antibacterial tests [19,20,21]. Different concentrations of ZnO–SiO_2_-pH5.0, ZnO–SiO_2_-pH7.0, and pure ZnO were added to the agar medium, and then *E. coli* (CGMCC 1.2385) was inoculated on the medium to observe the growth of bacteria and determine the minimum inhibitory concentration (MIC) [22].

The antimicrobial coating was obtained by mixing 12 wt % styrene-acrylic emulsion, 34 wt % of H_2_O, 50 wt % of the filler (0–8 wt % of ZnO–SiO_2_-pH7.0), and 4 wt % of paint additive. The antibacterial property of ZnO–SiO_2_ was evaluated by testing the antibacterial property of the coating. The antibacterial rate of the coating was tested according to Chinese national standard GB/T21866-2008 [23]. The antibacterial rate (R) is calculated as:

R = 100% × (*A* − *B*)/*A*, (2)

where *A* and *B* are the average number of colonies of the blank control plate and antibacterial coating plate after 24 h.

## 3. Results and Discussion

### 3.1. Structure and Characterization of ZnO–SiO_2_

#### 3.1.1. Phase and Chemical Constitution of ZnO–SiO_2_

**Figure 3:**
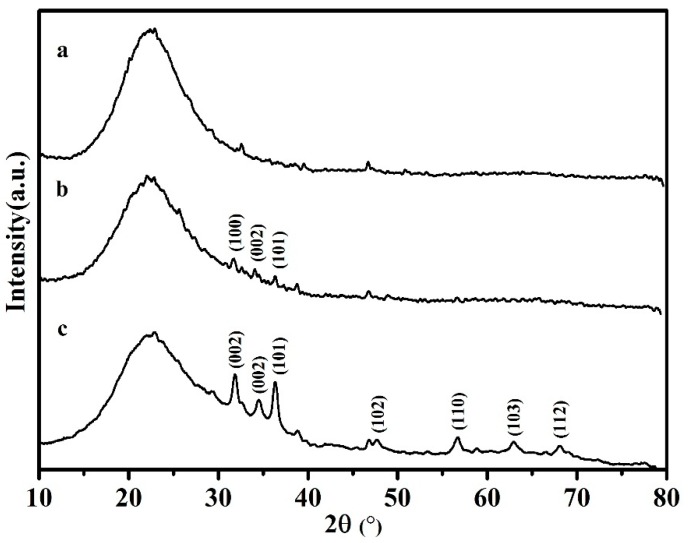
XRD of (**a**) amorphous SiO_2_; (**b**) ZnO–SiO_2_-pH5.0; and (**c**) ZnO–SiO_2_-pH7.0.

Figure 3 shows the XRD patterns of ZnO–SiO_2_. Table 1 shows the XRF results of each sample. The XRD pattern of the SiO_2_ carrier shows a strong bread peak near 2*θ* of 23°, indicating that the main phase is an amorphous phase corresponding to amorphous SiO_2_. In the XRD patterns of ZnO–SiO_2_-pH5.0 and ZnO–SiO_2_-pH7.0, in addition to the above-mentioned peak reflecting the amorphous phase, the peaks at 31.8°, 34.5°, 36.3°, and 47.5° correspond to the ZnO diffraction peak [24,25], indicating that Zn^2+^ has been transformed into ZnO after the thermal reaction with the SiO_2_ carrier and calcination. The ZnO diffraction intensity of ZnO–SiO_2_-pH7.0 was significantly larger than that of ZnO–SiO_2_-pH5.0 due to the different loadings of ZnO. The contents of ZnO in ZnO–SiO_2_-pH5.0 and ZnO–SiO_2_-pH7.0 are respectively 4.51 and 11.26% (Table 1). The SiO_2_ content in ZnO–SiO_2_-pH5.0 is lower than that in ZnO–SiO_2_-pH7.0. The results are consistent with the XRD results.

According to the XRD data in Figure 3, the grain size of ZnO–SiO_2_-pH7.0 was calculated to be 3.63 nm according to the Scherer Equation.

#### 3.1.2. Microstructure of ZnO–SiO_2_

**Figure 4:**
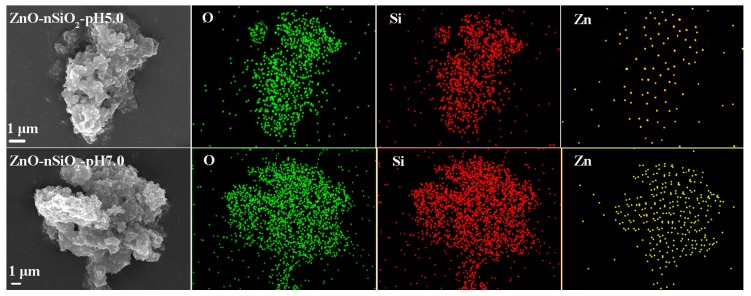
SEM images of ZnO–SiO_2_ and corresponding mapping results.

Figure 4 shows the distribution of three elements (O, Si, and Zn) in ZnO–SiO_2_-pH5.0 and ZnO–SiO_2_-pH7.0. The distributions of these three elements are consistent with the distribution of ZnO–SiO_2_ particles. The distribution densities of O and Si are larger than that of Zn. The Zn density in the elemental distribution of ZnO–SiO_2_-pH7.0 is greater than that of ZnO–SiO_2_-pH5.0. The results indicate that the main components of ZnO–SiO_2_ are SiO_2_. The content of ZnO is low, but evenly distributed on the surface of SiO_2_ particles.

**Figure 5:**
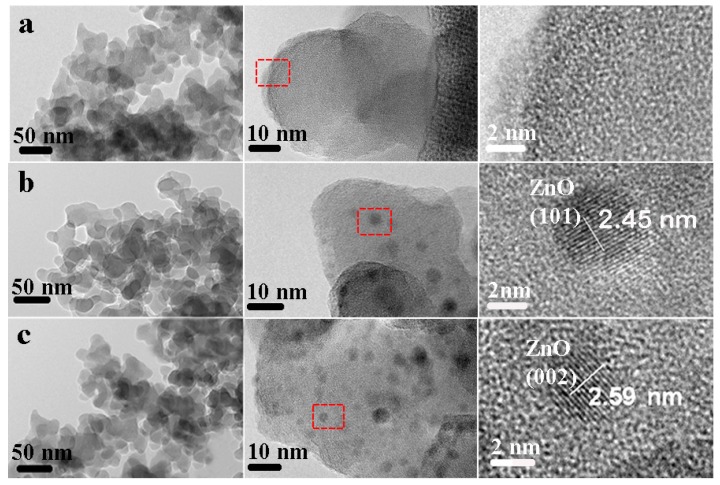
TEM maps of (**a**) amorphous SiO_2_; (**b**) ZnO–SiO_2_-pH5.0; (**c**) ZnO–SiO_2_-pH7.0 at different scales.

Figure 5 shows the TEM images of the amorphous SiO_2_ carrier, ZnO–SiO_2_-pH5.0, and ZnO–SiO_2_-pH7.0. At a small scale, all the samples are regular particle aggregates. The particle size is about 20–30 nm. Although these particles overlap each other, the overall dispersion effect is good. These unit particles are obviously amorphous SiO_2_ particles. At the scale of 10 nm, the surface morphology of SiO_2_ particles in the SiO_2_ carrier is uniform, indicating that no other material is loaded. At the scale of 2 nm, only homogeneous non-crystal phase particles are observed. Dark spots with a size of 3–8 nm are uniformly distributed in ZnO–SiO_2_-PH5.0 and ZnO–SiO_2_-pH7.0 at the scale of 10 nm. These dark spots are crystal phase particles at the larger magnification. The stripe spacing can reflect the lattice size. The stripe spacing of ZnO–SiO_2_-pH5.0 and ZnO–SiO_2_-pH7.0 are respectively measured to be 2.45 and 2.59 nm. ZnO (101) plane spacing and ZnO (002) plane spacing are respectively measured to be 2.45 and 2.59 Å, which are almost consistent with standard ZnO (101) plane spacing of 2.47 Å and ZnO (002) plane spacing of 2.60 Å (ICDD card # 89-7102). These data indicate that these ZnO nanoparticles were monodispersedly loaded on the SiO_2_ surface. The size of ZnO particles is 3–8 nm, which is consistent with the average particle size of 3.63 nm obtained in the XRD test [18].

#### 3.1.3. Formation Mechanism of ZnO–SiO_2_

**Figure 6:**
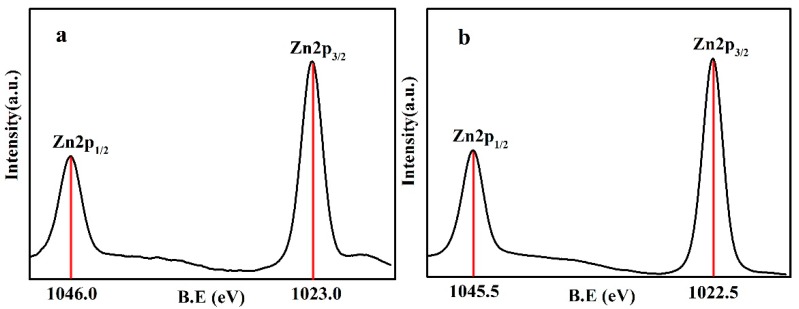
XPS of (**a**) ZnO–SiO_2_-pH5.0; (**b**) ZnO–SiO_2_-pH7.0.

Figure 6 shows the XPS pattern between 1015 and 1050 eV of the ZnO–SiO_2_. The peaks of ZnO–SiO_2_-pH5.0 and ZnO–SiO_2_-pH7.0 at 1046.0 and 1045.5 eV correspond to Zn2p_1/2_ orbital; the peaks at 1023.0 and 1022.5 eV correspond to the Zn2p_3/2_ orbital [26]. These peaks are equivalent to the 2p_1/2_ and 2p_3/2_ energy peaks (1044.2 and 1021.2 eV) of ZnO [27]. Moreover, the energy difference between Zn2p_1/2_ and Zn2p_3/2_ orbitals is 23 eV, which is the same as that of ZnO. Therefore, it can be determined that the valence of Zn in ZnO–SiO_2_-pH5.0 and ZnO–SiO_2_-pH7.0 is +2, which is consistent with the results of XRD, XRF, and TEM.

Table 2 shows the percentages of the amorphous SiO_2_ carrier, composite precursors (pH 5.0 and 7.0) and ZnO–SiO_2_ based on XPS. Compared with the amorphous SiO_2_ carrier, the Zn^2+^ composite precursors show the increasing ratio of O/Si with the increase in the Zn content. The change may be interpreted as follows. Hydroxyl groups generated by the hydrolysis of Zn form hydrogen bonds on the SiO_2_ surface, thus resulting in an increase in the amount of O. Compared with the precursors, ZnO–SiO_2_ products show a decreased O/Si ratio because the amount of oxygen is decreased by the dehydration condensation reaction among the –OH bonds on the surface of precursors during calcination. The above analysis suggests that ZnO nanoparticles was loaded on the surface of amorphous SiO_2._

**Figure 7:**
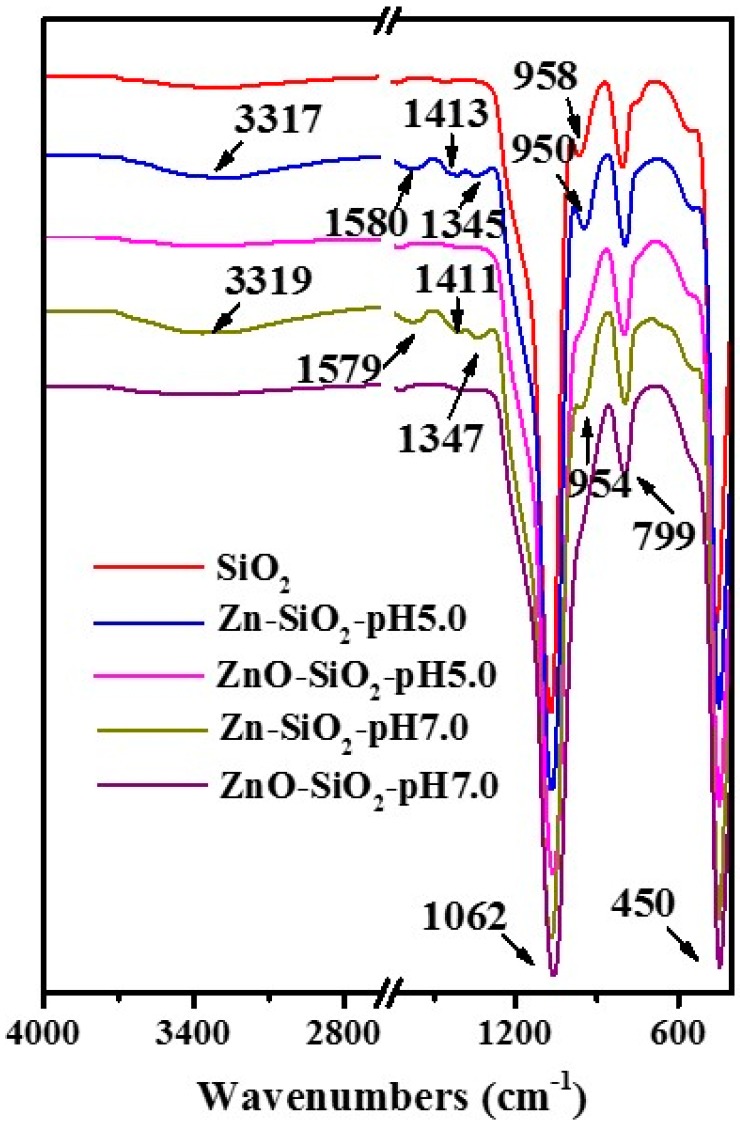
FTIR spectra of amorphous SiO_2_, Zn–SiO_2_-pH5.0, ZnO–SiO_2_-pH5.0, Zn–SiO_2_-pH7.0, and ZnO–SiO_2_-pH7.0.

Figure 7 shows the infrared spectra of the amorphous SiO_2_ carrier, Zn^2+^ composite precursor (Zn–SiO_2_-pH5.0 and Zn–SiO_2_-pH7.0), and the final products (ZnO–SiO_2_-pH5.0 and ZnO–SiO_2_-pH7.0). The absorption peaks of each sample at 450 and 1062 cm^−1^ are respectively ascribed to symmetrical and antisymmetric stretching vibration of Si–O–Si. The absorption peak at 799 cm^−1^ corresponds to bending vibration, reflecting the characteristics of SiO_2_ [28]. As shown in Figure 7, the infrared spectra of Zn^2+^ composite precursors (Zn–SiO_2_-pH5.0 and Zn–SiO_2_-pH7.0) at 3317 and 3319 cm^−1^ correspond to Zn–OH stretching vibration. The absorption peaks of hydroxyl groups in zinc hydroxide (Zn(OH)_2_) at 1345 and 1347 cm^−1^ reflect the bridging effect of the hydroxyl group in the product, and the Si–OH bending vibration peak in the two products moves from 958 cm^−1^ (the vibration peak of the raw material SiO_2_) to 952 and 954 cm^−1^, respectively. The changes indicate that in the hydrothermal reaction during the preparation process of Zn–SiO_2_-pH5.0, Zn^2+^ forms a complex of Zn(OH)_2_, which yields hydrogen bonds with Si–OH on the surface of amorphous SiO_2_ [29,30,31]. In addition, the –OH bending vibration peaks of water adsorbed on the surface of Zn–SiO_2_-pH5.0 and Zn–SiO_2_-pH7.0 occur at 1413 and 1411 cm^−1^, respectively. The –OH shear vibration peaks occur at 1580 and 1579 cm^−1^ [32]. The Zn–OH and Si–OH of calcined products and the –OH bond of adsorbed water disappeared after the calcination of the precursors. Therefore, the high-temperature calcination resulted in the evaporation of SiO_2_ surface water and the dehydration condensation reaction between Si–OH and Zn–OH, and yielded Si–O–Zn chemical bond.

**Figure 8:**
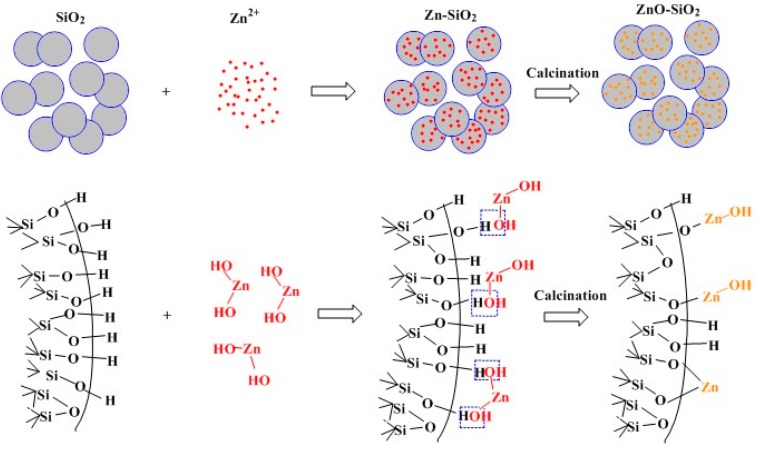
Synthesis of the ZnO–nSiO_2_ Complex

Figure 8 shows a schematic diagram of the synthesis process of ZnO–SiO_2_ by loading Zn^2+^ on the amorphous SiO_2_ carrier via the hydrothermal reaction and composite precursor calcination. Due to the large number of Si–OH bonds on the surface of SiO_2_ and the strong activity of Si–OH bonds, the interaction between Si–OH and Zn–OH is greater than the interaction in Zn–OH (multinuclear ions). Therefore, Zn^2+^ is immobilized on the surface of SiO_2_ and dispersed ZnO nanoparticles are formed after the calcination of precursors.

### 3.2. Antibacterial Properties of ZnO–SiO_2_

**Figure 9:**
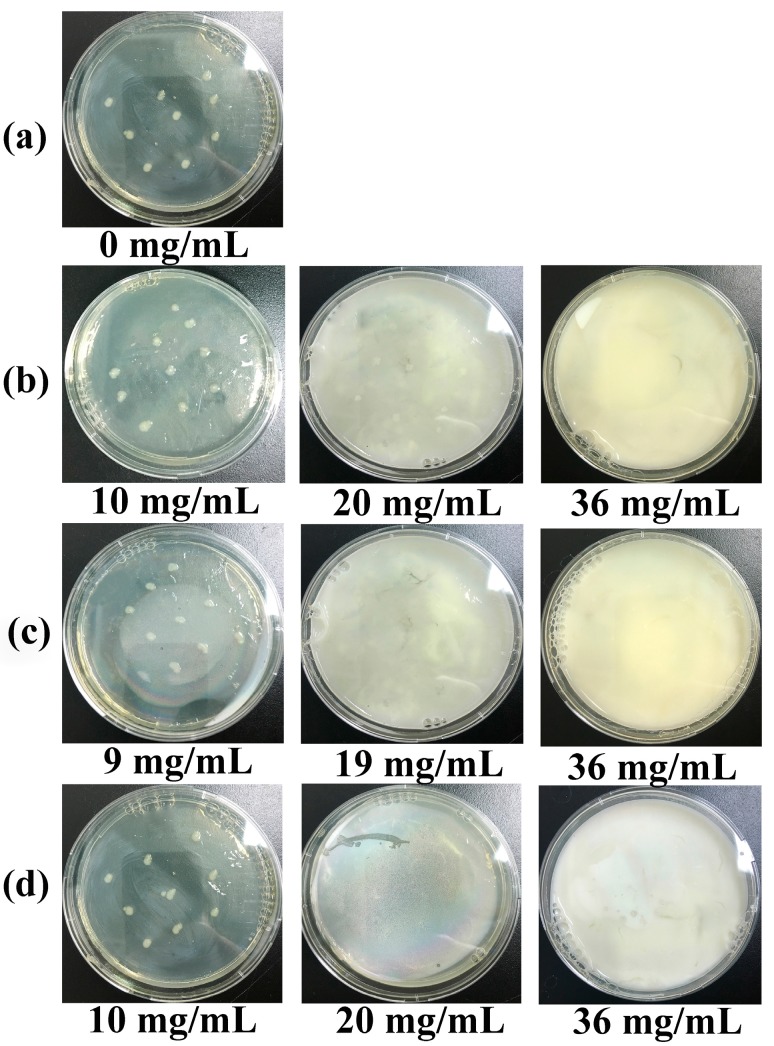
Antimicrobial tests of (**a**) blank control, (**b**) different concentrations of ZnO–SiO_2_-pH5.0 in agar medium, (**c**) different concentrations of ZnO–SiO_2_-pH7.0 in agar medium, (**d**) different concentrations of pure ZnO in agar medium.

ZnO has antibacterial activity under light and dark conditions and is mostly applied under dark conditions. In order to investigate the antibacterial properties of ZnO–SiO_2_ under dark conditions, ZnO–SiO_2_-pH5.0, ZnO–SiO_2_-pH7.0, and pure ZnO were respectively prepared. Figure 9 shows the bacterial growth profiles obtained by the plate test. Obvious colonies were formed in the blank control without the antimicrobial material (Figure 9a). When the concentration of ZnO–SiO_2_-pH5.0 was 10 mg/mL, obvious colonies were observed on the culture plate; when the concentration of ZnO–SiO_2_-pH5.0 was 20 mg/mL, the number of colonies decreased but colonies did not completely disappear; when the concentration of ZnO–SiO_2_-pH5.0 was increased to 36 mg/mL, no colony was formed (Figure 9b). The concentrations of ZnO–SiO_2_-pH7.0 and pure ZnO required for colony-free results were respectively 19 mg/mL and 20 mg/mL (Figure 9c,d). Based on the above results, the minimum inhibitory concentration (MIC) of each sample was determined and converted into the minimum inhibitory concentration of ZnO based on the content of ZnO in the composite (Table 3). The MIC values of ZnO–SiO_2_-pH5.0 and ZnO–SiO_2_-pH7.0 were respectively 1.60 and 2.14 mg/mL, which were equivalent to 10% of the MIC of pure ZnO (20 mg/mL), indicating that the antimicrobial ability of ZnO nanoparticles loaded on the SiO_2_ surface was about 10 times that of pure ZnO. Obviously, the formation of dispersed nanoparticles (3–8 nm) loaded on amorphous SiO_2_ greatly improved its antimicrobial performance.

**Figure 10:**
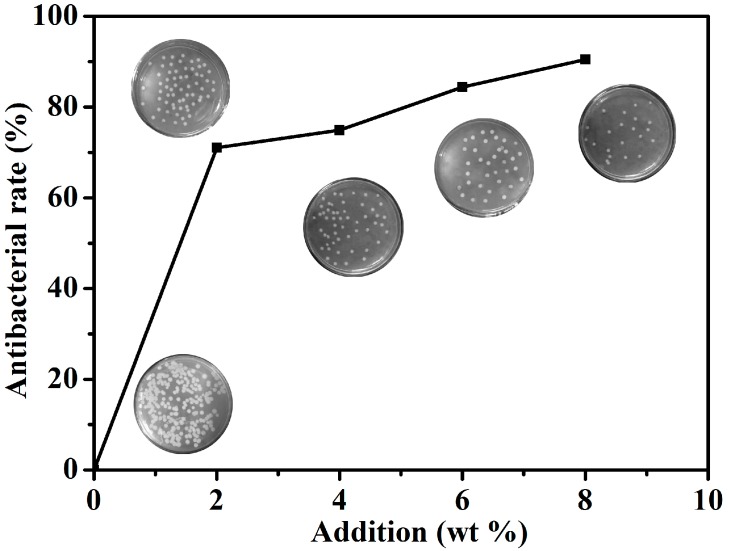
Effects of different additives on antibacterial rate (*E. coli*).

Figure 10 shows the antibacterial rate and colony growth conditions of *E. coli* on the plates added with different amounts of ZnO–SiO_2_-pH7.0 coating. The antibacterial rate of the coating without ZnO–SiO_2_-pH7.0 was 0 and a large number of colonies were formed on the plate, indicating that the coating showed no antibacterial property. When the addition of ZnO–SiO_2_-pH7.0 in the coating was only 2%, the antibacterial rate was increased above 70%, showing a good antibacterial effect; when the dosage was gradually increased to 8%, the antibacterial rate of the coating to *E. coli* was 90.48%, which met the requirements of the antibacterial effect of antibacterial coating in Chinese national standard GBT21866-2008. The increasing antibacterial rate of the paint indicated less colonies and a better antibacterial effect.

The antimicrobial properties of the ZnO nanoparticles supported uniformly and dispersedly on the surface of SiO_2_ were greatly improved, due to the large specific surface area and surface activity of ZnO nanoparticles compared with the pure ZnO of large particles, and the contact and inhibition with microbes is stronger. This should be considered as one of the means to enhance the function of ZnO.

## 4. Conclusions

ZnO–SiO_2_ composite was prepared by an environmentally friendly hydrothermal method and high-temperature calcination. In this composite, ZnO nanoparticles with a particle size of 3–8 nm were uniformly and dispersedly loaded on the surface of amorphous SiO_2_. The size of the ZnO particles used in the industry is about 500 nm, and there is a certain degree of agglomeration among particles. According to the analysis of relevant tests, the strong interaction between the SiO_2_ carrier surface and Zn–OH (precursors of ZnO) reduced the combination between Zn–OH, prevented its aggregation and formed monodispersed nanoparticles. Compared with pure ZnO, ZnO–SiO_2_ showed much better antibacterial performance in the MIC test and the characterization test of paint properties.

In general, ZnO nanoparticles loaded uniformly and depressively on the surface of amorphous SiO_2_ greatly enhanced its antibacterial function.

## Figures and Tables

**Table 1:** XRF of amorphous SiO_2_, ZnO–SiO_2_-pH5.0 and ZnO–SiO_2_-pH7.0.

Samples	SiO_2_/%	ZnO/%	Na_2_O/%
amorphous SiO_2_	96.63	0	0.98
ZnO–SiO_2_-pH5.0	92.78	4.51	1.05
ZnO–SiO_2_-pH7.0	84.22	11.26	2.68

**Table 2:** Element analysis based on XPS results

Samples	C1s (%)	Zn2p (%)	Si2p (%)	O1s (%)	O/Si
Amorphous SiO_2_	2.74	0	31.27	65.84	2.11
Zn–SiO_2_-pH5.0	6.18	2.24	27.55	62.04	2.25
ZnO–SiO_2_-pH5.0	3.48	2.47	30.03	63.68	2.12
Zn–SiO_2_-pH7.0	6.90	6.24	24.38	60.84	2.50
ZnO–SiO_2_-pH7.0	5.96	10.55	25.18	58.39	2.31

**Table 3:** MIC of ZnO–SiO_2_-pH5.0, ZnO–SiO_2_-pH7.0, and pure ZnO.

MIC (mg/mL)	ZnO–SiO_2_-pH5.0	ZnO–SiO_2_-pH7.0	ZnO
*E. coli*	36	19	20
*E. coli* (ZnO)	1.60	2.14	20
